# Case report: Renal clear cell carcinoma presenting with metastatic nodules in the male breast: a case and literature review

**DOI:** 10.3389/fimmu.2026.1729630

**Published:** 2026-03-30

**Authors:** Yangcai Wang, Jia Li, Jiang Zhao, Ji Zheng, Zhenqiang Fang, Bishao Sun

**Affiliations:** 1 Department of Urology, Second Affiliated Hospital, Army Medical University, Chongqing, China; 2 Department of Medicinal Chemistry, College of Pharmacy, Army Medical University, Chongqing, China

**Keywords:** breast metastasis, case report, clear cell renal cell carcinoma, literature review, renal cell carcinoma

## Abstract

Metastasis of renal cell carcinoma (RCC) most frequently occurs in the lungs, bones, liver, and brain. Breast metastasis from RCC is exceedingly rare, particularly in male patients presenting with a breast mass as the initial manifestation. We report a 54-year-old man who presented with a breast nodule that, upon excision, was pathologically confirmed as metastatic Clear cell RCC. Abdominal computed tomography (CT) revealed a primary lesion in the left kidney. One month and nine months after laparoscopic partial nephrectomy, recurrent metastatic lesions were detected in the breast. After two subsequent excisions of the breast metastases, the patient was treated with lenvatinib in combination with tislelizumab. However, treatment was discontinued due to intractable diarrhoea. Three months after treatment cessation, follow-up CT imaging revealed widespread systemic metastases. The patient is currently maintained on sunitinib monotherapy. The total duration of follow-up was 30 months. This case, together with a review of earlier reported cases of RCC with breast metastasis, reveals the interesting observation that female patients with RCC appear to be more susceptible to “delayed” breast metastasis compared with male patients. Our analysis underscores the need for thorough evaluation of breast nodules in patients with an RCC history, even absent classic signs like nipple retraction or discharge. These findings may contribute to the future development of effective management guidelines for breast metastases from RCC and help clinicians better recognize this clinical entity.

## 1 Introduction

Renal cell carcinoma (RCC) is one of the common malignant tumours of the urinary system. Clear cell renal cell carcinoma (ccRCC) accounts for approximately 80% of renal cell carcinoma cases, representing the most prevalent histological subtype (1). Early-stage renal cancer is often asymptomatic, with most patients diagnosed incidentally through imaging studies revealing a renal mass. The classic triad of advanced renal cancer includes haematuria, flank pain, and an abdominal mass (2). RCC typically metastasizes via hematogenous and/or lymphatic routes to nearly any organ in the body. Common sites of metastasis and local recurrence include the lungs, bones, liver, brain, and regional lymph nodes (3). Metastatic renal cell carcinoma to the breast is exceedingly rare, with only a limited number of documented cases in the literature, and often poses a diagnostic challenge, leading to frequent misdiagnosis. We herein report a case of a male patient with ccRCC who initially presented with a breast nodule. Following partial nephrectomy of the left kidney, he experienced recurrent metastases in the right breast region. After resection of the breast metastases, the patient received combined targeted and immunotherapy. However, treatment was discontinued due to adverse drug effects. A re-evaluation three months after cessation revealed widespread metastases involving the left kidney, left retroperitoneum, lungs, pancreatic head and iliac vessels. The patient remains alive 30 months after initial diagnosis. Furthermore, we conducted an analysis based on 48 previously reported cases of RCC with breast metastasis, aiming to consolidate the current understanding and improve clinical recognition of this rare entity.

## 2 Case presentation

**Figure 1:**
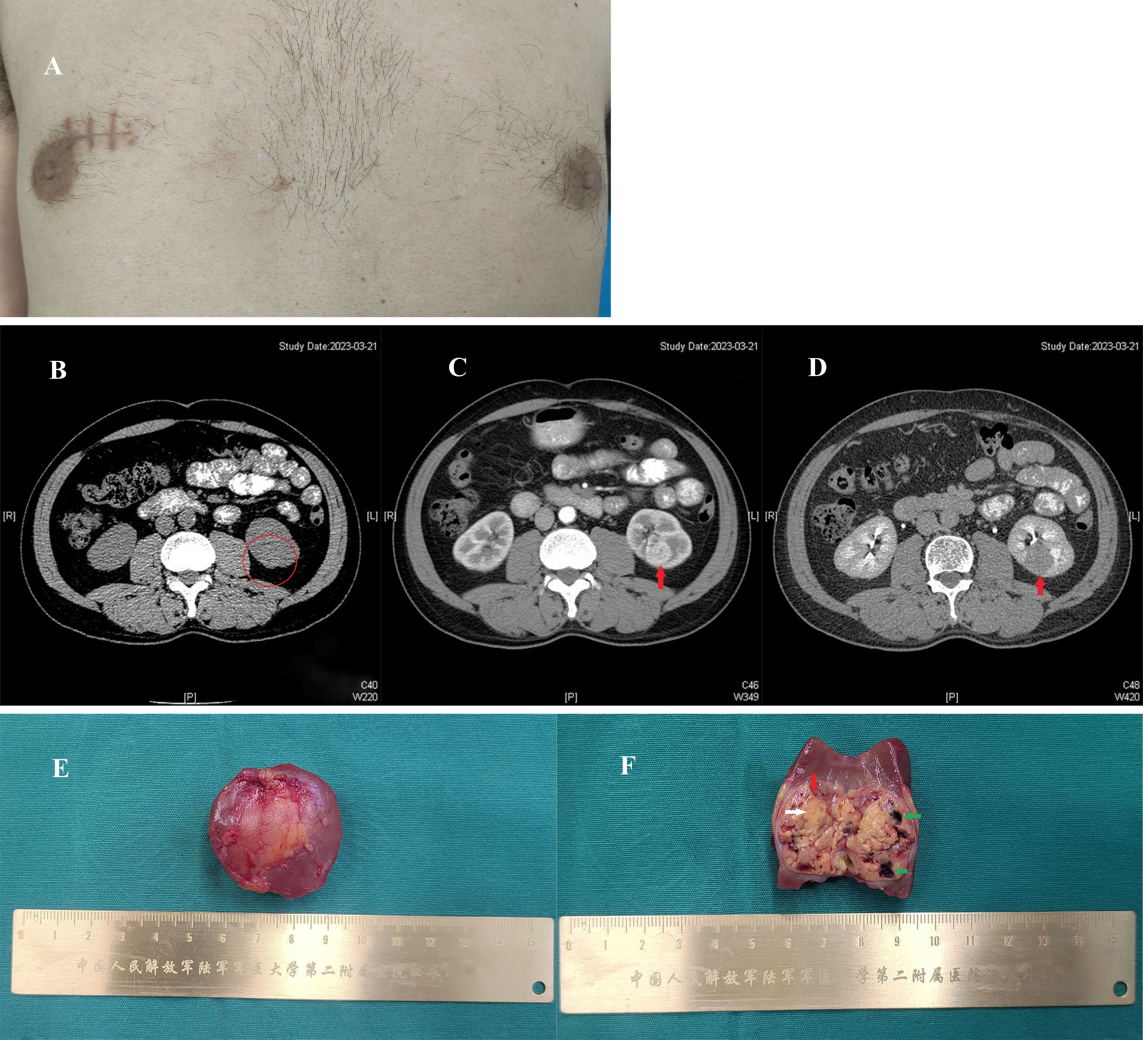
Postoperative view of the right breast after lumpectomy, dynamic contrast-enhanced CT features of the left renal mass and gross morphology of the resected left renal tumor. **(A)** A well-healed, approximately 2-cm linear scar is noted in the upper inner quadrant of the right breast. The surrounding skin shows normal coloration, with no palpable mass or signs of recurrence. **(B)** Non-contrast phase: A mass (circled in red) in the lower pole of the left kidney is iso-dense to the renal parenchyma with ill-defined margins. **(C)** Corticomedullary phase (enhanced): The mass (red arrow) in the posterior aspect of the lower pole shows well-defined margins and avid, heterogeneous enhancement. **(D)** Excretory phase: The mass (red arrow) demonstrates rapid contrast washout, becoming less dense than the persistently enhancing normal renal parenchyma. **(E)** Tumor tissue with partial renal tissue, measuring approximately 3 cm in diameter. **(F)** Sectioned surface of the tumor; the tumor is predominantly golden yellow (white arrow), with focal fibrous strands (red arrow) and hemorrhage (green arrow) observed locally.

**Figure 2:**
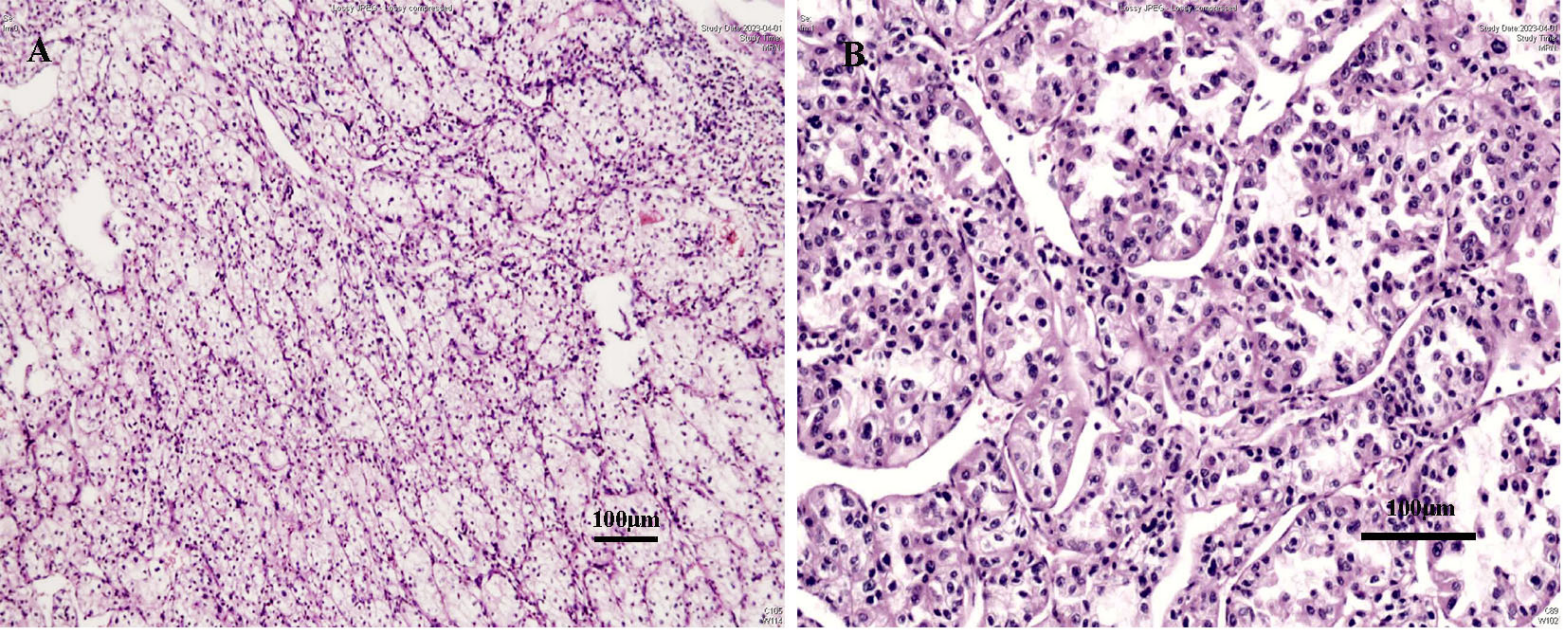
Histopathological features of ccRCC in the left renal tumor. **(A)** At low magnification (×200), the tumor demonstrates a characteristic nested (*alveolar*) architecture, surrounded by a prominent network of branching, thin-walled blood vessels (*scale bar: 100 μm*). **(B)** At high magnification (×400), the tumor cells exhibit abundant eosinophilic (pink) cytoplasm and round nuclei with distinct nucleoli, typical of ccRCC (*scale bar: 100μm*).

A 54-year-old, non-smoking man (body mass index 21.61 kg/m²) with no family history of malignancy presented in March 2023 following the incidental discovery of a right breast nodule. Following local excision at an external hospital, pathology confirmed ccRCC with clear margins. He was subsequently referred to our institution. Physical examination revealed a surgical scar adjacent to the right breast, measuring approximately 2 cm in length (**Figure 1A**), with no palpable nodules. Abdominal CT revealed a 3.0 cm tumour at the lower pole of the left kidney. The dynamic contrast-enhanced characteristics (non-contrast, corticomedullary, and excretory phases) are shown in **Figures 1B–D**, respectively. PET/CT showed no other metastatic lesions. On March 27, 2023, he underwent a retroperitoneal laparoscopic partial nephrectomy. Macroscopic examination of the specimen showed a golden-yellow tumour with areas of fibrosis and haemorrhage (**Figures 1E, F**). Histopathology confirmed left-sided ccRCC, WHO/ISUP grade 2, staged as IV (T1aN0M1) according to the AJCC 8th edition (**Figure 2**).

**Figure 3:**
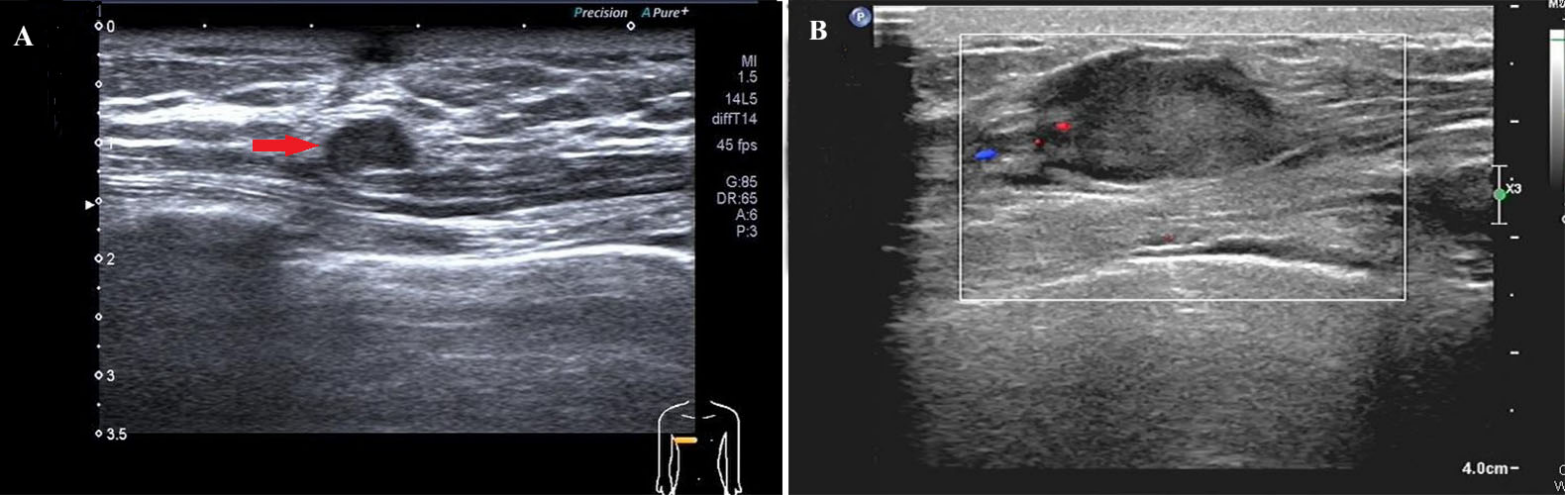
Ultrasound of the breast mass in the breast region. **(A)** Right breast ultrasound (April 2023): A small nodule in the right breast, measuring 0.88 cm × 0.54 cm (indicated by the red arrow). **(B)** Right breast ultrasound (December 2023): A nodule in the right breast, measuring 2.32 cm × 2.35 cm (within the rectangular frame).

**Figure 4:**
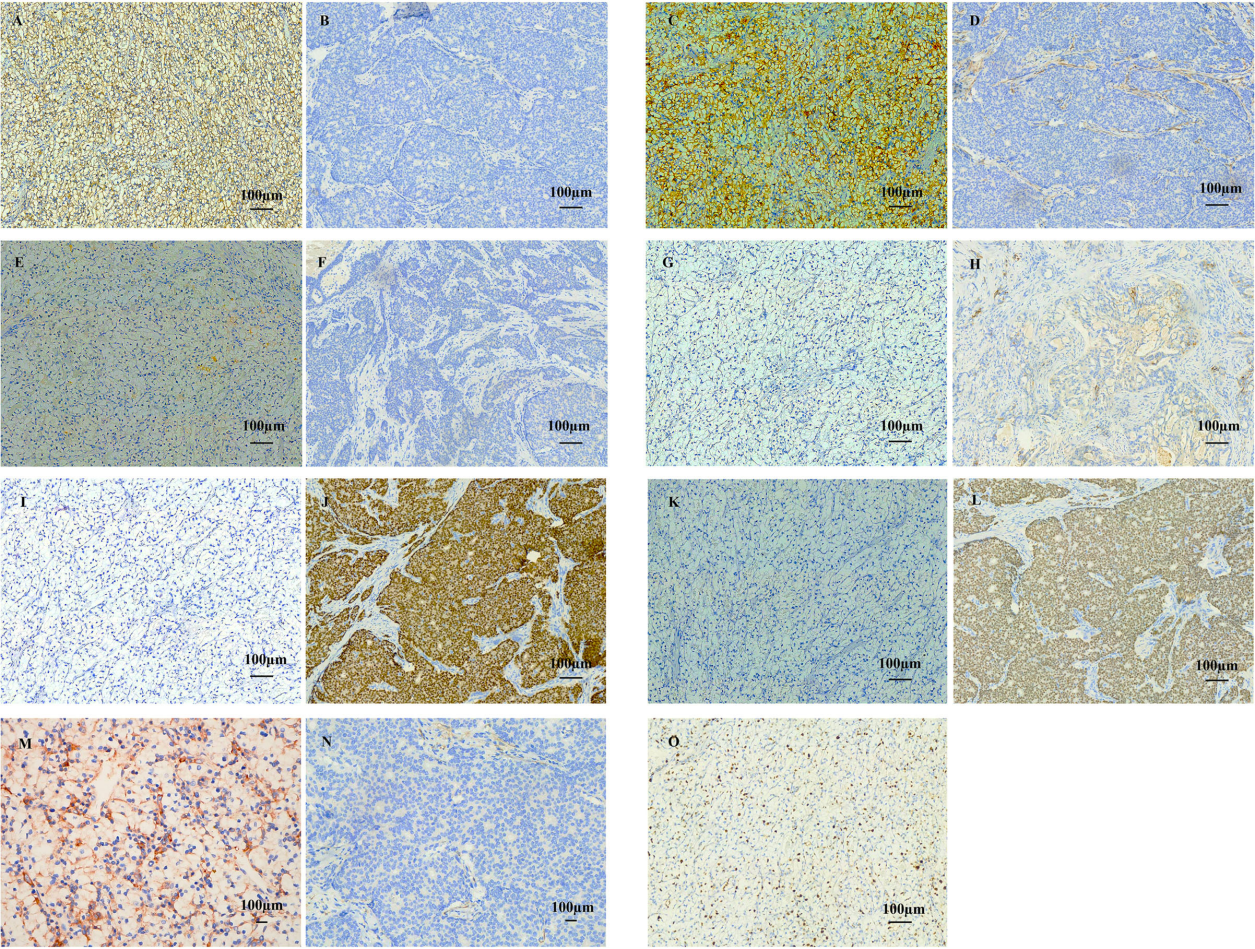
Immunohistochemical profile confirming metastatic clear cell renal cell carcinoma (ccRCC) and excluding primary breast carcinoma. **(A)** CA9: Strong and diffuse membranous staining in tumor cells (scale bar: 100 μm). **(B)** CA9 (control): Negative in control breast cancer cells (scale bar: 100 μm). **(C)** CD10: Diffuse membranous and luminal staining in tumor cells (scale bar: 100 μm). **(D)** CD10 (control): Negative in control breast cancer cells (scale bar: 100 μm). **(E)** PAX8: Strong nuclear positivity in a subset of tumor cells (scale bar: 100 μm). **(F)** PAX8 (control): Negative in control breast cancer cells (scale bar: 100 μm). **(G)** Mammaglobin: Negative in tumor cells, consistent with non-breast origin (scale bar: 100 μm). **(H)** Mammaglobin (control): Scattered cytoplasmic positivity in control breast cancer cells (scale bar: 100 μm). **(I)** GATA3: Negative in tumor cells, excluding primary breast carcinoma (scale bar: 100 μm). **(J)** GATA3 (control): Diffuse nuclear positivity in control breast cancer cells (scale bar: 100 μm). **(K)** PR: Negative in tumor cells, arguing against hormone receptor-positive breast cancer (scale bar: 100 μm). **(L)** PR (control): Diffuse nuclear positivity in control breast cancer cells (scale bar: 100 μm). **(M)** PD-L1: Positive in tumor cells (TPS ≥1%; 22C3 antibody, DAKO Link 48 platform) (scale bar: 100 μm). **(N)** PD-L1 (control): Negative in control breast cancer cells (scale bar: 100 μm). **(O)** Ki-67: Proliferation index approximately 20% in tumor cells (scale bar: 100 μm).

In April 2023, the patient palpated a recurrent mass in the right breast. Ultrasound demonstrated a 0.88 cm × 0.54 cm nodule (**Figure 3A**). A right total mastectomy was performed on April 18, 2023. Immunohistochemical analysis (**Figure 4**) of the mastectomy specimen showed a profile positive for PAX8 (focal), CD10, CA9, and PD-L1 and negative for GATA3, progesterone receptor (PR), and mammaglobin, confirming a locally recurrent metastatic ccRCC with clear margins. However, the patient declined further treatment due to personal financial constraints and was subsequently managed with clinical surveillance only.

In December 2023, a new nodule was noted in the right mastectomy scar. Ultrasound identified a 2.32 cm × 2.35 cm subcutaneous nodule (**Figure 3B**), suggestive of metastasis. Wide local excision was performed, and histopathology confirmed recurrent ccRCC with clear margins.

Following recovery from surgery and resolution of his financial constraints, the patient initiated systemic therapy with lenvatinib (18 mg orally once daily) plus tislelizumab (200 mg intravenously every three weeks) for 10 cycles. During treatment, serial CT assessments demonstrated stable disease (SD) in all target lesions according to RECIST 1.1 criteria and this stability was maintained over approximately 10 treatment cycles. Treatment was discontinued in October 2024 due to adverse drug effects, including severe diarrhoea and rash; the diarrhoea was characterised by approximately 11 watery bowel movements daily accompanied by moderate dehydration and electrolyte disturbances, necessitating hospitalisation for intravenous fluid repletion, and was graded as Grade 3 according to CTCAE v5.0 criteria.

In March 2025, follow-up CT of the chest and abdomen was performed. Imaging revealed a newly developed mass at the previous surgical site of the left lower renal pole, along with newly identified metastatic foci in the left lower lung lobe, the right anterosuperior chest wall musculature, the pancreatic head, the retroperitoneum, and adjacent to the left iliac vessels. According to RECIST 1.1 criteria, these findings constituted progressive disease (PD).

For this progression, second-line therapy with sunitinib was initiated in March 2025 at a starting dose of 50 mg daily (4 weeks on/2 weeks off). During treatment, the patient developed grade 2 diarrhoea (approximately three episodes per day), leading to a dose reduction to 37.5 mg daily for toxicity management. A follow-up CT scan performed in June 2025 (approximately 3 months after treatment initiation) for response assessment showed a reduction in the sum of diameters of the target lesions in the left kidney and retroperitoneum compared with the pretreatment baseline in March 2025, with no new lesions observed. According to RECIST 1.1 criteria, this was assessed as stable disease (SD). The patient continues on maintenance sunitinib at this dose and remains clinically stable.

## 3 Discussion

The majority (50–60%) of RCC cases are diagnosed incidentally via non-invasive imaging, while 30–50% present with metastatic disease at initial diagnosis (4). Hematogenous dissemination is the predominant route of metastasis, resulting from tumour cell invasion into the kidney’s abundant venous network. Additionally, lymphatic spread may occur, typically involving the renal hilar and retroperitoneal lymph nodes initially, with potential progression to mediastinal nodes and subsequent systemic dissemination via the thoracic duct. RCC may also spread by direct local invasion, independent of vascular or lymphatic channels, through contiguous growth into adjacent structures. In the present case, PET-CT revealed no metastases beyond the breast, and abdominal CT showed no enlarged retroperitoneal lymph nodes. Thus, hematogenous spread is the most plausible route of breast metastasis.

The prevalence of breast metastases from extramammary malignancies remains poorly defined, with a reported incidence ranging from 0.1% to 5.0% (5). The most common primary tumours metastasizing to the breast include melanoma, haematological malignancies (e.g., leukaemia and lymphoma), lung cancer, and ovarian cancer (6). Metastasis of RCC to the breast is exceptionally rare, and comprehensive clinical data are limited. To comprehensively characterize the clinical profile of breast metastases from RCC, a systematic literature search was performed in the PubMed database for articles published between January 1, 1990, and October 11, 2025. The following Boolean search string was used:(“Carcinoma, Renal Cell”[Mesh] OR “Kidney Neoplasms”[Mesh] OR renal cell carcinoma[tiab] OR RCC[tiab] OR kidney cancer*[tiab]) AND (“Neoplasm Metastasis”[Mesh] OR metastas*[tiab] OR secondary[tiab]) AND (“Breast Neoplasms”[Mesh] OR breast[tiab] OR mammary[tiab]).

The initial search yielded 1327 records. After sequential screening, 46 articles reporting on 48 patients were included. Combined with our case, we analysed the clinical features of 49 patients with RCC metastatic to the breast. The clinicopathological features of this cohort are summarized in **Table 1**. The detailed clinicopathological data for all 49 individual cases are provided in **Supplementary Table 1**.

**Table 1:** Summary of clinicopathological characteristics and outcomes in 49 cases of RCC metastatic to the breast.

Characteristic	Findings
Total Cases	49 (Including the present case and 48 from literature)
Age	Median: 65 years (Range: 14–88)
Sex	Female: 46 (93.9%); Male: 3 (6.1%)
Laterality of Breast Metastasis	Unilateral: 41 (Right: 22, Left: 19); Bilateral: 3; Not specified: 5
Key IHC Profile	Positive: PAX8 (most frequent), CD10, Vimentin. Negative: GATA3, CK7
Primary RCC Histology	Clear cell RCC (ccRCC): 27 (55.1%); Undescribed Type of RCC (utRCC): 18; Papillary RCC: 2; Not specified: 2
Metastasis Interval	Range: Synchronous to 20 years after nephrectomy; Synchronous presentation: 14 cases (28.6%)
Primary Management	Local surgical excision: 31 (63.3%); Systemic therapy*: 15 (30.6%); Supportive/Palliative care: 4
Clinical Outcome	Favorable† (NED/Stable/Response): 23 (46.9%); Poor‡ (Death/Widespread progression): 12 (24.5%); Not reported: 14 (28.6%)

*Systemic therapy includes tyrosine kinase inhibitors (e.g. sunitinib, pazopanib), immunotherapy (e.g. interleukin-2, checkpoint inhibitors), interferon, or chemotherapy.

†Favorable: No evidence of disease, stable disease, or partial/complete response.

‡Poor: Death, or widespread metastatic progression.

IHC, immunohistochemistry; RCC, renal cell carcinoma; ccRCC, clear cell RCC; utRCC, unspecified type RCC.

Including the current case, 49 cases are available for analysis. Patient ages ranged from 14 to 88 years, with 97.96% of patients over 18 years and approximately 91.67% over 50 years. No cases of breast metastasis from RCC have been reported in young children, which may be attributable to underdeveloped breast tissue reducing the likelihood of hematogenous seeding. Among the 49 patients, only 28 (57.1%) had a history of renal cancer surgery. The interval between nephrectomy and breast metastasis diagnosis ranged from 5 months (7) to 20 years (8). Notably, only one case (3.57%) had an interval of less than one year. In contrast, intervals exceeding three, five, and ten years were observed in 22 (78.57%), 17 (60.71%), and 7 (25.00%) cases, respectively.

Among the 49 patients, only two were male (9, 10), both presenting with unilateral breast metastasis as the initial manifestation, synchronously diagnosed with the primary RCC. Our patient also presented with a breast mass as the initial symptom, consistent with previous reports in males. Interestingly, he underwent three surgical excisions for recurrent ccRCC breast metastases. Despite negative margins in all procedures, recurrence occurred, possibly due to residual micrometastatic disease in the breast region. Of the 49 cases, unilateral breast metastasis was reported in several patients, with ipsilateral (7, 11–26) and contralateral (8, 9, 27–41) involvement occurring at similar frequencies relative to the primary renal tumour. This suggests that RCC metastasizes to either breast with equal probability, supporting hematogenous rather than direct lymphatic spread.

Regarding histological subtypes, only two cases of papillary RCC (7, 42) were reported, while 26 were ccRCC, the remaining cases lacked subtype specification. This distribution reflects the higher overall incidence of ccRCC compared to other subtypes.

Prognosis in patients with unilateral breast metastasis from RCC depends largely on whether the breast lesion represents an isolated metastasis. Patients with isolated breast metastases who underwent complete excision achieved disease-free survival of up to three years (14, 17), with potential for longer overall survival. In contrast, patients with additional extra-mammary metastases at diagnosis had poorer outcomes (13, 29). Those with bilateral breast metastases (42–44) also exhibited worse prognosis compared to those with unilateral involvement. For instance, a 14-year-old patient with bilateral breast metastases and widespread disease at diagnosis (42) had an unreported survival duration, while two other bilateral cases survived only six months (42, 43). Bilateral breast involvement likely indicates a higher tumour burden and portends a poorer prognosis.

Most breast metastases from RCC present as a palpable mass without skin involvement, nipple retraction, or discharge. Rarely, they may manifest as rapidly growing, painless masses (18, 22). Although these features can help distinguish them from primary breast cancer, misdiagnosis remains common. One reported case involved a woman with a history of breast cancer who was initially diagnosed with local recurrence, yet subsequent histology revealed metastatic RCC, leading to the identification of the primary renal tumour (15). Fine-needle aspiration cytology (FNAC) and core needle biopsy (CNB) are commonly used in diagnosing breast lesions; however, both carry a risk of misdiagnosis in breast metastases from RCC, with FNAC exhibiting higher false-negative rates (9, 10, 16, 17, 20, 32, 34, 40, 42, 45, 46). When metastasis is suspected, excisional biopsy with histopathological examination remains the most reliable diagnostic approach (17, 32, 35, 37, 47). Immunohistochemistry plays a critical role in distinguishing metastatic RCC from primary breast carcinoma, with markers such as PAX8, CD10, CK7, Vimentin, and RCC Ma being particularly useful (48–50). A history of RCC is an important clue for pathologists and should always be communicated to aid accurate diagnosis (8, 12, 51–53).

The treatment landscape for advanced RCC has evolved to include targeted therapy combined with immunotherapy or dual immunotherapy regimens (54). Among the 46 cases with available data, adjuvant therapies included interleukin-2 or interferon (3–6, 8–23, 27–38, 43–45, 47, 51, 55), targeted therapy (18, 30, 35), and combined targeted and immune therapy (12). While these regimens provided short-term disease control in some cases, follow-up was generally limited, and long-term outcomes were not reported.

The management of our case was guided by the principles of dynamic assessment and stepwise treatment escalation, which are central to the personalized therapy of advanced RCC (56). Following the initial resection of a breast metastasis in April 2023, the patient presented with a small ipsilateral nodule (<1 cm) within a short interval. Given that this was still consistent with oligometastatic RCC, a total mastectomy was therefore undertaken as a more extensive secondary resection for the solitary lesion, aiming for local disease control (57). Regrettably, although adjuvant systemic therapy was recommended, it was deferred by the patient due to financial constraints. A third recurrence (lesion >2 cm) occurred only 8 months after the mastectomy. This early recurrence highlighted the tumour’s aggressive biology and suggested that local excision alone was inadequate for disease control, marking a transition from a “potentially locally controllable” state to an “active progression phase necessitating systemic intervention.” The patient was classified as having intermediate-risk disease according to the International Metastatic RCC Database Consortium (IMDC) criteria. The breast metastasis was PD-L1-positive on immunohistochemistry. Despite the higher toxicity profile of combination targeted and immune therapy compared to monotherapy (58), its superior efficacy led to the initiation of lenvatinib plus tislelizumab once the patient’s financial situation allowed (59). Serial CT assessments during this combination regimen showed stable disease (RECIST 1.1 criteria).

Treatment was discontinued due to adverse effects. The rapid systemic progression observed 3 months later underscores the ‘disease flare’ or rebound effect associated with treatment interruption in RCC, a phenomenon previously reported (60) and potentially linked to a surge in endothelial cell proliferation (61). Although recent prospective trials indicate that a planned treatment break may be feasible in selected responders (62), our case differs critically: the interruption was unplanned, and the disease had demonstrated an aggressive, rapidly recurring phenotype prior to systemic therapy. This further reflects the aggressive nature of the breast metastases. Considering the prior toxicity and the patient’s financial limitations, a low-dose sunitinib maintenance regimen was initiated (63, 64). He has now been followed for 30 months, exceeding the follow-up duration in 93.88% of reported cases.

In summary, this case illustrates several key points: Breast metastases from RCC require a highly individualized approach. Early recurrence after adequate local therapy signifies local treatment failure and represents the optimal trigger for systemic therapy. Repeated local recurrences may indicate residual regional micrometastatic disease. While combination therapies have advanced RCC management, toxicity remains a challenge, and vigilance is needed for potential disease rebound upon treatment cessation—a mechanism requiring further study.

A persisting challenge in RCC is the lack of validated molecular biomarkers for both screening and monitoring (65), leaving reliance on radiographic surveillance. Although potential biomarkers have been reported (66–68), they await validation in randomized clinical trials.

## 4 Conclusion

Breast metastases from RCC are rare and diagnostically challenging, frequently mimicking primary breast cancer. We report a case of synchronous RCC metastasis to the breast, in which the breast lesion was the initial clinical manifestation. Existing literature reveals distinct epidemiological patterns: while such metastases in male patients are often synchronous and unilateral, in female patients they typically present after the age of 50 and are characteristically delayed, emerging 3–10 years following nephrectomy for RCC. Although diagnostic confirmation usually requires excision biopsy, the management of an isolated metastasis may combine surgical resection with early systemic therapy. This case highlights that any new breast nodule in an RCC patient should raise suspicion for metastasis, even in the absence of the classic “delayed” presentation or typical breast cancer signs. This distinction is critical, as it redirects the diagnostic workup and substantially alters therapeutic strategy.

## Data Availability

The original contributions presented in the study are included in the article/**Supplementary Material**. Further inquiries can be directed to the corresponding authors.
