# Supplementary material for: Case report: Renal clear cell carcinoma presenting with metastatic nodules in the male breast: a case and literature review
Source: Front Immunol. 2026 Mar 30;17:1729630. doi: 10.3389/fimmu.2026.1729630 (PMC13071389; doi:10.3389/fimmu.2026.1729630)
Supplement: Supplementary file 1 [file DataSheet1.pdf]

Table S1: The detailed clinicopathological data for all 49 individual cases(*The reference numbers in this table correspond to the reference list provided at the end of this supplementary file*.)

First Author, Year	Patient Age/Sex	Metastasis Interval	Type of RCC (size)	Clinical Presentation	Diagnostic Method	IHC	Management	Outcome
Our case	54/M	Synchronous	ccRCC (3.0cm)	Incidentally found to have a right breast nodule	Excision of the nodule and	-	Laparoscopic partial nephrectomy; lenvatinib and tislelizumab for 10 months; maintained on sunitinib monotherapy	Alive at 30 months
Sahoo[1], 2025	54/F	6 years	Not Specified	Regular imaging found a mass in (0.6cm) lower outer quadrant right breast	CNB	PAX8+ CD10+ MNF116+	Wide local excision; no systemic therapy	Stable postoperatively
Breese[2], 2023	68/F	20 years	Left utRCC	Screening mammography found a mass (0.6cm) in upper outer right breast	Biopsy	PAX8+ GATA3-	Partial mastectomy; no systemic therapy	Not Specified
Pernicone[3], 2023	76/F	Synchronous	Left ccRCC	Screening mammography found a mass (0.25cm) in medial left breast	CNB	PAX8+ RCC+ CD10+	Pembrolizumab and Lenvatinib	Complete resolution of metastases and 50% reduction in primary tumou at 6 months
Spasic[4], 2023	82/F	11 years	Right utRCC	Palpable in right upper quadrants breast mass (2.0cm)	CNB	CD10+ EMA+ Vimentin+	Partial breast resection	No disease progression at 17 months
Elouarith[5], 2022	69/F	18 years	Right ccRCC	Poorly limited and irregular mass (2.5 cm p) in upper outer quadrant left breast	Biopsy	PAX8+ CD10+ GATA3- CK7-	Lumpectomy	Not specified
Khurram[6], 2021	65/F	Synchronous	Right ccRCC	4-month history of rapidly growing right sided breast lump.	US-guided CNB	CD117+ PAX-8+ Vimentin+	Supportive care	Succumbed to raised intracranial pressure
Ali[7], 2021	73/F	3 years	Left ccRCC (18.0cm)	Newly palpable, hard, poorly mobile right breast mass (1-2 cm)	US-guided CNB	PAX8+ CK7- GATA3-	Palliative care	Widespread metastases poor prognosis
Nguyen[8], 2020	57/F	Prior history (unspecified)	Left utRCC	Screening mammography found mass (1.0cm) in posterior upper inner quadrant of right breast	US-guided CNB	PAX8+	Sunitinib (TKI therapy)	Most metastatic lesions regressed; ongoing treatment
Ikarashi[9], 2018	57/F	2 years	Right ccRCC	Asymptomatic; 0.8 cm enhancing left breast mass on surveillance CT	CNB	CD10+	Segmental resection	NED at 1-year follow-up
Amadu[10], 2018	75/F	4 years	Right utRCC	Incidental, tiny, palpable, mobile nodule in left breast	Inconclusive CNB; Surgical excision	Vimentin+ CD10+	Metastasectomy	NED at 1-year follow-up
Xu[11], 2017	68/F	10 years	ccRCC	incidental discovery of a nodule (1.0cm) in her left breast	Surgical excision	CD10+ Vimentin+ ER- HER2- PR-	Surgical excision	No recurrence at 2-month follow-up with no systemic therapy
Ishigaki[12], 2017	82/ F	9 years	Left ccRCC	Asymptomatic; 1 cm mass in the lateral portion of the right breast on surveillance CT	CNB	Not Specified	Partial mastectomy	No evidence of disease at 18 months
Dhamoon[13], 2017	63/F	5 years	Right ccRCC (5.0cm)	Asymptomatic; Annual screening found a mass (4.0cm) between the lower quadrants of the left breast	CNB	Vimentin+ CD10+	Lumpectomy + sentinel lymph node biopsy	Chose watchful waiting; NED at 1-year follow-up
Koch[14], 2016	55/F	8 years	Left ccRCC	Screening mammogram found a mass in the lower outer quadrant of the left	Biopsy	CK7+ CD10+	Lumpectomy	NED at 3-year follow-up

				breast				
Chen[15], 2014	74/F	1 year	Right ccRCC	A 2-millimeter lesion was identified in the postoperative biopsy specimen, primarily surrounded by invasive ductal carcinoma.(Right)	Modified radical mastectomy	Vimentin+ CD10+	Modified radical mastectomy	Alive at 1-year follow-up with the use of tamoxifen and interferon therapy. No systemic therapy of ccRCC
Solaini[16], 2014	44/F	Synchronous	Left utRCC (8.0cm)	Solitary, painless, smooth, mobile left breast mass (1.5cm)	Inconclusive FNAC	E-cadherin+	Quadrantectomy and palliative care	Widespread metastases at diagnosis; died 4 months later
Saluja[17], 2014	57/M	Synchronous	Right ccRCC (10.1cm)	A palpable right breast mass (1.3cm)	Inconclusive FNAC and Excisional biopsy	CK7+ CD10+ PAX-8+ CK+	Right nephrectomy	NED at 3-year follow-up
Botticelli[18], 2013	60/F	4 years	Right ccRCC (5.4cm)	Screening mammogram found a nodule in the lower inner quadrant of left breast	Inconclusive FNAC and Surgical pathology	Vimentin+ CD10+	Surgery for excision of the nodule	Not specified
Pathe[19], 2012	64/F	10 years	Left ccRCC	A continuously increasing right breast lump (2.7cm)	Surgical pathology	Not Specified	Pazopanib as systemic therapy	Alive at 4-month follow-up
Mahrous[20] 2012	58/F	5 years and 7 months	Left ccRCC (7.0cm)	A painless, palpable, and rapidly growing left breast mass (4.0cm)	Fine needle aspiration biopsy	Vimentin+	Breast lump Excision and Systemic therapy of sunitinib	Survived for 14 months after breast lump excision
Balliauw[21], 2011	83/F	Synchronous	Left utRCC	New well-marginated opacity in right breast on routine follow-up (prior breast CA history)	FNAC CNB	CD10+ CK7- CK20-	No oncological therapy	Progressive growth of breast mass at 8 months; stable other metastatic sites
Durai[22], 2009	68/F	Synchronous	Left ccRCC	New lump (2.0cm) in contralateral left breast (prior breast CA history)	US-guided CNB	Vimentin+ CD10+	Not specified	Not specified
Hairulfaizi[23], 2009	67/F	5 years	Right ccRCC	Two right breast masses with ipsilateral axillary lymph node	Inconclusive FNAC and Mastectomy	CD10+ CK7+ CK20-	Mastectomy with axillary clearance; no systemic therapy	NED at 2-year follow-up
Daneshbod[24], 2008	65/F	8 years	Right ccRCC	Painless, enlarging, solitary right breast mass	Inconclusive FNAC and Excisional biopsy	Not Specified	Excisional biopsy	Not specified
Bortnik[25], 2008	55/F	2 years and 10 months	Left ccRCC (5.9cm)	Screening mammography found a mass (0.6cm) in right breast	Excisional biopsy	Not Specified	Metastasectomy; no systemic therapy	NED at 2.5-year follow-up
Ganapathi[26], 2008	88/F	4 years	ccRCC	Bilateral breast lumps	CNB	Not Specified	Left mastectomy and excision of the right breast lump	Alive at 6-month follow-up
Alzaraa[27], 2007	81/F	5.5 years	Right utRCC	Incidental discovery of a right breast lump (1.7cm)	CNB	Not Specified	Metastasectomy; no systemic therapy	Declined interferon; under regular follow-up at time of report
Ding[28], 2007	50/F	Not Specified	utRCC	Palpable right breast lump	FNAC	CD10+ CK7-	Wide local excision; not specified of systemic therapy	Died 2 months post-operatively with disseminated disease (brain, lung, liver)
Lee[29], 2007	71/F	Synchronous	Right ccRCC (16cm)	Rapidly enlarging right breast mass with abdominal discomfort	CNB	CD10+	Interleukin-2 immunotherapy	Solitary cerebral metastasis resected and alive at 5- months follow-up
McLaughlin[30], 2006	76/F	12 years	Right ccRCC (5.0cm)	Enlarging, solitary, painless, left-sided breast mass (1.1cm)	Surgical excision pathology	Not Specified	Lumpectomy; subcutaneous IL-2	Alive at 3-month follow-up
Gacci[31], 2005	79/F	3 years	Right ccRCC (4.5cm)	Nodule in right breast	Excisional biopsy	Not Specified	Lumpectomy; adjuvant interferon therapy	Alive 10 months after surgery
Smymiotis[32], 2005	65/M	Synchronous	Left utRCC	Suspicious hard palpable mass 3 x 3 cm	FNAC not diagnostic; Mastectomy pathology	Not Specified	Left nephrectomy	Not Specified
O'Sullivan[33],	52/F	Synchronous	utRCC	Right breast mass	FNAC and	Vimentin+	Radical nephrectomy &	Outcome not

2003				identified during workup for abdominal symptoms	biopsy	CK+	thrombectomy; metastasectomy	specified
Vassalli[34], 2001	72/F	9 years	Left utRCC	A nodular lesion in the lateral superior quarter of the left breast;	FNAC and lumpectomy	Not Specified	Lumpectomy; no systemic therapy	Disease-free at follow-up
Gupta[35], 2001	31/F	5 months	Left Papillary RCC	Left breast mass	Excisional biopsy	S100- CD31+ CD34+	Not Specified	Not Specified
Grossklaus[36], 2000	77/F	Synchronous	Right ccRCC (8.0cm)	Screening mammogram found a mass (2.5cm) in right breast	Excisional biopsy of the breast lesion	Not Specified	Right radical nephrectomy	Not Specified
Chhieng[37], 1999	39-78 / F (3 cases)	Not Specified	utRCC	Solitary, painless breast mass (~1 cm) in all three cases	FNAC	Not Specified	Not Specified	Outcome not specified
Forte[38], 1999	71/F	6 years	Left ccRCC	A discrete lump (10cm) in the right breast	Radical mastectomy biopsy	Not Specified	Radical mastectomy	Outcome not specified
Kannan[39], 1998	65/F	Synchronous	Not Specified	A 2-cm mass in the upper outer quadrant of the right breast	Inconclusive FNAC; Surgical excision biopsy	Not Specified	Not Specified	Not Specified
Heggarty[40], 1998	63/F	Synchronous	Left utRCC (10.0cm)	Discrete masses (2.0cm) were palpable in both breasts	FNAC; Post-operative histopathology	EMA+ CAM5.2+	Interferon therapy	Alive at 6-month follow-up
Pursner[41], 1997	14/F	1 year	Right Papillary RCC	Bilateral palpable breast masses	Inconclusive FNAC; Diagnostic excisional biopsy	Not Specified	Bilateral metastatic excision	Presented with widespread metastases (bone, abdomen)
Bowditch[42], 1996	62/F	15 years	Right utRCC	Asymptomatic, impalpable mass (1.0cm) in left breast	Inconclusive FNAC; Localization biopsy	Not Specified	Excision; medroxyprogesterone acetate therapy	Developed skin metastasis at 5 months
Ferrara[43], 1996	57/F	2 years	utRCC	2 cm nodule, upper outer quadrant, left breast	FNAC	Vimentin+ CK+	Excision of breast nodule; post-operative chemotherapy	Died of pulmonary metastases 18 months after diagnosi
Gibbons[44], 1995	73/M	Synchronous	Left utRCC	A mass in upper outer quadrant, right breast	Inconclusive FNAC; Mastectomy biopsy	Not Specified	Simple mastectomy; radiotherapy to chest wall	Nephrectomy not performed due to disseminated disease; outcome not specified
Lesho[45], 1992	74/F	Synchronous	Left ccRCC	A mass in the left breast	Post-operative histopathology	Not Specified	Modified radical mastectomy	Nephrectomy planned; outcome not specified
Masters[46], 1990	69/F	Synchronous	Left ccRCC	A discrete mobile lump in the upper outer quadrant of the right breast	Excisional biopsy	Not Specified	Wide local excision; subsequent nephrectomy	New contralateral breast lesion at 19 months; stable on oral progesterone

Abbreviations: F: Female; M: Male; Synchronous: The breast metastasis was diagnosed concurrently with the primary renal cell carcinoma. CNB:Core needle biopsy; IHC: Immunohistochemistry; FNAC: Fine-needle aspiration cytology; NED: No evidence of disease; CT: Computed Tomography; utRCC: Undescribed Type of RCC.

1. Sahoo AS, Singh B, Salman M, Ragad L, Elayyan R. Rare metastasis of renal cell carcinoma to the breast: a case report. Oxf Med Case Rep. (2025) 2025:omaf02. doi: 10.1093/omcr/omaf029
2. Breese RO, Friend K. Case report of renal cell carcinoma metastasis to the breast. Am Surg. (2023) 89:3541–2. doi: 10.1177/00031348231161711
3. Pernicone E, Fabrega-Foster K. Clinically silent, metastatic renal cell carcinoma detected on routine screening mammogram: a report of a rare case and review of literature. Cureus. (2023) 15:e48352. doi: 10.7759/cureus.48352
4. Spasic M, Zaric D, Mitrovic M, Milojevic S, Nedovic N, Sekulic M, et al. Secondary breast Malignancy from renal cell carcinoma: challenges in diagnosis and treatmentcase report. Diagnostics (Basel). (2023) 13:991. doi: 10.3390/diagnostics13050991
5. Elouarith I, Bouhtouri Y, Elmajoudi S, Bekarsabein S, Ech-Charif S, Khmou M, et al. Breast metastasis 18 years after nephrectomy for renal cell carcinoma: a case report. J Surg Case Rep. (2022) 2022:rjac116. doi: 10.1093/jscr/rjac116
6. Khurram R, Amir T, Chaudhary K, Joshi A, Nayagam K, Tincey S. Metastatic renal cell carcinoma initially presenting as a unilateral breast lump. Radiol Case Rep. (2021) 16:945–9. doi: 10.1016/j.radcr.2021.02.006
7. Ali HOE, Ghorab T, Cameron IR, Marzouk AMSM. Renal cell carcinoma metastasis to the breast: a rare presentation. Case Rep Radiol. (2021) 2021:6625689. doi: 10.1155/2021/6625689
8. Nguyen QD, Ko H, Robinson AS, Lee AE, He J. Renal cell carcinoma diagnosis after initial detection on screening mammogram. Cureus. (2020) 12:e10428. doi: 10.7759/cureus.10428
9. Ikarashi D, Ishida K, Kashiwaba M, Kato Y, Shiomi E, Takayama M, et al. Sporadic breast metastasis derived from renal cell carcinoma: a case report. Urol Case Rep. (2017)16:126–8. doi: 10.1016/j.eucr.2017.11.032
10. Amadu AM, Marras V, Crivelli P, Soro D, Conti M, Meloni GB. Isolated breast metastasis 4 years after nephrectomy. Breast J. (2018) 24:85–7. doi: 10.1111/tbj.12837

11. Xu Y, Hou R, Lu Q, Deng Y, Hu B. Renal clear cell carcinoma metastasis to the breast ten years after nephrectomy: a case report and literature review. Diagn Pathol. (2017) 12:76. doi: 10.1186/s13000-017-0666-8
12. Ishigaki T, Kinoshita S, Shimada N, Miyake R, Suzuki M, Takeyama H. Breast metastasis nine years after nephrectomy for renal cell carcinoma: a case report. Int J Surg Case Rep. (2017) 39:145–9. doi: 10.1016/j.ijscr.2017.08.008
13. Dhannoon SM, Alsaad AA, Asmar AR, Shahin FH. Renal cell carcinoma with isolated breast metastasis. BMJ Case Rep. 2017 Jun 15;2017:bcr2016219124. doi: 10.1136/bcr-2016-219124.
14. Koch A, Stoll F, Duclos B, Chenard MP, Mathelin C. Intramammary metastasis of renal cell carcinoma: a diagnostic trap. Gynecol Obstet Fertil. (2016) 44:128–9. doi: 10.1016/j.gyobfe.2015.12.005
15. Chen TD, Lee LY. A case of renal cell carcinoma metastasizing to invasive ductal breast carcinoma. J Formos Med Assoc. (2014) 113:133–6. doi: 10.1016/j.jfma.2012.07.022
16. Solaini L, Bianchi A, Filippini L, Lucini L, Simoncini E, Ragni F. A mammary nodule mimicking breast cancer. Int Surg. (2014) 99:200–2. doi: 10.9738/INTSURG-D-12-00019.1
17. Saluja K, Thakral B, Bit-Ivan E, Kaufman M, Liu L. Fine-needle aspiration of metastatic renal cell carcinoma to a male breast: a rare initial presentation. Cytojournal. (2014) 11:8. doi: 10.4103/1742-6413.131737
18. Botticelli A, De Francesco GP, Di Stefano D. Breast metastasis from clear cell renal cell carcinoma. J Ultrasound. (2013) 16:127–30. doi: 10.1007/s40477-013-0026-9
19. Pathe N, Raymond J, Cintra AU. Metastatic renal cell cancer presenting as a breast mass. Clin Adv Hematol Oncol. (2012) 10:124–6.
20. Mahrous M, Al Morsy W, Al-Hujaily A, Al-Sulimani S. Breast metastasis from renal cell carcinoma: rare initial presentation of disease recurrence after 5 years. J Breast Cancer. (2012) 15:244–7. doi: 10.4048/jbc.2012.15.2.244
21. Balliauw C, Termote B, Van Steen A, Moerman P, Christiaens MR, Van Ongeval C. Metastatic renal cell carcinoma presenting as a breast mass in a woman with history of primary breast cancer. JBR-BTR. (2011) 94:330–2. doi: 10.5334/jbr-btr.699
22. Durai R, Ruhomauly SN, Wilson E, Hoque H. Metastatic renal cell carcinoma presenting as a breast lump in a treated breast cancer patient. Singapore Med J. (2009)50:e277–9.
23. Hairulfaizi H, Rohaizak M, Naqiyah I, Yahya M, Tan G, Zainuddin Z. Breast and axillary lymph nodes metastasis five years after radical nephrectomy for renal cell carcinoma a case report and review of the literature. Libyan J Med. (2009) 4:120–2.doi: 10.4176/090510
24. Daneshbod Y, Khojasteh HN, Atefi S, Aledavood A. Renal cell carcinoma presenting as a solitary breast mass. a diagnostic pitfall on aspiration cytology of clear cell tumors of the breast. Breast J. (2008) 14:388–90. doi: 10.1111/j.1524-4741.2008.00606.x
25. Bortnik S, Cohen DJ, Leider-Trejo L, Ron IG. Breast metastasis from a renal cell carcinoma. Isr Med Assoc J. (2008) 10:736–7.
26. Ganapathi S, Evans G, Hargest R. Bilateral breast metastases of a renal carcinoma: a case report and review of the literature. BMJ Case Rep. (2008) 2008:bcr0620080239. doi: 10.1136/bcr.06.2008.0239
27. Alzaraa A, Vodovnik A, Montgomery H, Saeed M, Sharma N. Breast metastasis from a renal cell cancer. World J Surg Oncol. (2007) 5:25. doi: 10.1186/1477-7819-5-25
28. Ding GT, Hwang JS, Tan PH. Sarcomatoid renal cell carcinoma metastatic to the breast: report of a case with diagnosis on fine needle aspiration cytology. Acta Cytol. (2007) 51:451–5. doi: 10.1159/000325765
29. Lee WK, Cawson JN, Hill PA, Hoang J, Rouse H. Renal cell carcinoma metastasis to the breast: mammographic, sonographic, CT, and pathologic correlation. Breast J. (2007) 13:316–7. doi: 10.1111/j.1524-4741.2007.00433.x
30. McLauglin SA, Thiel DD, Smith SL, Wehle MJ, Menke DM. Solitary breast mass as initial presentation of clinically silent metastatic renal cell carcinoma. Breast. 2006 Jun;15(3):427-9. doi: 10.1016/j.breast.2005.09.009
31. Gacci M, Orzalesi L, Distante V, Nesi G, Vezzosi V, Livi L, Mungai V, et al. Renal cell carcinoma metastatic to the breast and breast cancer metastatic to the kidney: two rare solitary metastases. Breast J. (2005)11:351–2. doi: 10.1111/j.1075-122X.2005.00057.x
32. Smymiotis V, Theodosopoulos T, Marinis A, Goula K, Psychogios J, Kondi-Pafiti A. Metastatic disease in the breast from nonmammary neoplasms. Eur J Gynaecol Oncol. (2005) 26:547–50.
33. O'Sullivan AW, Kelly PM, Smith JM, Gorey TF. Renal cell carcinoma metastasis to breast. Ir J Med Sci. (2003) 172:48. doi: 10.1007/BF02914791
34. Vassalli L, Ferrari VD, Simoncini E, Rangoni G, Montini E, Marpicati P, et al. Solitary breast metastases from a renal cell carcinoma. Breast Cancer Res Treat. (2001) 68:29–31. doi: 10.1023/a:1017990625298
35. Gupta D, Merino MI, Farhood A, Middleton LP. Metastases to breast simulating ductal carcinoma in situ: report of two cases and review of the literature. Ann Diagn Pathol. (2001) 5:15–20. doi: 10.1053/adpa.2001.21476
36. Grossklaus DJ, Holzbeierlein JM, Roth BJ, Smith JA. Abnormal mammogram as the presenting sign of renal cell carcinoma. J Urol. (2000) 163:1239–40. doi: 10.1097/00005392-200004000-00041
37. Chhieng DC, Cohen JM, Waisman J, Fernandez G, Skoog L, Cangiarella JF. Fineneedle aspiration cytology of renal-cell adenocarcinoma metastatic to the breast: a report of three cases. Diagn Cytopathol. (1999) 21:324–7. doi: 10.1002/(sici)1097-0339(199911)21:5<324::aid-dc5>3.0.co;2-q
38. Forte A, Peronace MI, Gallinaro LS, Bertagni A, Prece V, Montesano G, et al. Metastasis to the breast of a renal carcinoma: a clinical case. Eur Rev Med Pharmacol Sci. (1999) 3:115–8. doi: 10.1201/9781003032106-3
39. Kannan V. Fine-needle aspiration of metastatic renal-cell carcinoma masquerading as primary breast carcinoma. Diagn Cytopathol. (1998) 18:343–5. doi: 10.1002/(sici)1097-0339(199805)18:5<343::aid-dc7>3.0.co;2-b
40. Heggarty P, McCusker G, Clements WD. Bilateral breast metastases from a renal carcinoma. Int J Clin Pract. (1998) 52:443–4. doi: 10.1111/j.1742-1241.1998.tb08902.x
41. Pursner M, Petchprapa C, Haller JO, Orentlicher RJ. Renal carcinoma: bilateral breast metastases in a child. Pediatr Radiol. (1997) 27:242–3. doi: 10.1007/s002470050111
42. Bowditch MG, Peck R, Shorthouse AJ. Metastatic renal adenocarcinoma presenting in a breast screening programme. Eur J Surg Oncol. (1996) 22:641–3. doi: 10.1016/s0748-7983(96)92632-3
43. Ferrara G, Nappi O. Metastatic neoplasms of the breast: fine-needle aspiration cytology of two cases. Diagn Cytopathol. (1996) 15:139–43. doi: 10.1002/(SICI)1097-0339(199608)15:2<139::AID-DC12>3.0.CO;2-I
44. Gibbons CE, Lewi HJ, Kashif KM. Breast lump–an unusual presentation of renal cell carcinoma. Br J Urol. (1995) 76:131. doi: 10.1111/j.1464-410x.1995.tb07846.x
45. Lesho EP. Metastatic renal cell carcinoma presenting as a breast mass. Postgrad Med. (1992) 91:145–6. doi: 10.1080/00325481.1992.11701351
46. Masters A. Hypernephroma presenting as a lump in the breast. Aust N Z J Surg.(1990) 60:305–6. doi: 10.1111/j.1445-2197.1990.tb07372.x
